# Written narratives from immigrants following a prenatal diagnosis: qualitative exploratory study

**DOI:** 10.1186/s12884-019-2292-9

**Published:** 2019-05-06

**Authors:** Tommy Carlsson, Banaz Balbas, Elisabet Mattsson

**Affiliations:** 1 Sophiahemmet University, Box 5605, SE-114 86 Stockholm, Sweden; 2 Department of Women’s and Children’s Health, Uppsala University, Uppsala, Sweden; 3 Department of Health Care Sciences, Ersta Sköndal Bräcke University College, Stockholm, Sweden

**Keywords:** Consumer health information, Counselling, Immigrants, Prenatal diagnosis, Support

## Abstract

**Background:**

Expectant parents often have optimistic expectations of the obstetric ultrasound examination and are unprepared for a diagnosis of foetal anomaly. Research that gives voice to the experiences of immigrants faced with a prenatal diagnosis is scarce, and there is a need for more exploratory research that provides insights into the experiences of these persons. The aim of this study was to explore narratives of experiences of immigrants with Arabic or Sorani interpreter needs when presented with a prenatal diagnosis of foetal anomaly.

**Methods:**

A web-based tool with open-ended questions was distributed via Arabic and Kurdish non-profit associations and general women’s associations in Sweden. Responses were received from six women and analysed with qualitative content analysis.

**Results:**

The analysis resulted in three themes: (1) an unexpected hurricane of emotions, (2) trying to understand the situation though information in an unfamiliar language, and (3) being cared for in a country with accessible obstetric care and where induced abortion is legal.

**Conclusions:**

Immigrant women described an unexpected personal tragedy when faced with a prenatal diagnosis of foetal anomaly, and emphasised the importance of respectful and empathic psychological support. Their experiences of insufficient and incomprehensible information call attention to the importance of tailored approaches and the use of adequate medical interpreting services. There is a need for more descriptive studies that investigate decision-making and preparedness for induced abortion among immigrants faced with a prenatal diagnosis.

**Electronic supplementary material:**

The online version of this article (10.1186/s12884-019-2292-9) contains supplementary material, which is available to authorized users.

## Background

In many parts of the world, obstetric ultrasound examinations have been implemented as routine procedures to determine gestational length and number of foetuses, localise the placenta, and assess the anatomy of the foetus to screen for foetal anomalies. This routine procedure has led to an increase in the total number of foetal anomalies detected [1]. The total prevalence of congenital anomalies is approximately 24 per 1000 births, the most common being congenital heart defects, limb defects, and chromosomal anomalies [2].

Expectant parents often have optimistic expectations of the obstetric ultrasound examination, regard it as an important step towards parenthood, and perceive it as an opportunity for a visual confirmation of the pregnancy [3]. Research among native speakers has shown that a prenatal diagnosis of a foetal anomaly involves various psychological consequences, such as psychological distress and acute grief reactions [4]. Depending on legislative rights to induced abortion, expectant parents may be presented with the alternative to terminate the pregnancy following the diagnosis. Providing information about prenatal diagnosis that leads to an informed decision on whether to continue or terminate the pregnancy is challenging [5], especially in an intercultural context [6]. Factors influencing a woman’s decision include the severity of the malformation, socioeconomic status [7], prenatal attachment [6], psychological status [5], and religious beliefs [5, 8].

Expectant parents who continue the pregnancy express fears related to the risk of pre- or postnatal loss of the expected child [9], experience increased prenatal attachment as the pregnancy progresses [10], and show high levels of psychological distress after the birth [11]. Those who terminate the pregnancy regard the decision as burdensome, and experience the abortion as an emotionally and physically painful procedure [12]. Moreover, a substantial number of women show high levels of psychological distress months to years later, although few report feelings of regret [13].

Following a prenatal diagnosis of foetal anomaly, expectant parents experience a need for detailed and honest information about various topics. When presented with the option to legally terminate the pregnancy, they seek information in order to reach, affirm and come to terms with a decision to continue or terminate the pregnancy [14]. Expectant parents who continue the pregnancy seek detailed information during the course of pregnancy to enable them to feel sufficiently prepared for the birth and postpartum period [15]. Those who terminate the pregnancy express a need for preparatory information about various topics related to the actual procedure as well as the time that follows the abortion [16]. Consequently, information is a key aspect in the care of expectant parents faced with a prenatal diagnosis, regardless of whether the pregnancy is continued or terminated. However, many desire additional consultations with health professionals [7] and consider the medical information from health professionals complex [14].

It is estimated that 10% of the European population are immigrants, and many of these are women of reproductive age [17]. In comparison with natives, immigrants are at a higher risk of adverse pregnancy outcomes [18], report less satisfaction with maternity care services [19], and may face communication barriers during consultations with health professionals [18, 19]. However, research that gives voice to the experiences of immigrants faced with a prenatal diagnosis is scarce, and there is a need for more explorative research that provides insights into the experiences of these persons. The aim of this study was to explore narratives of experiences of immigrants with Arabic or Sorani interpreter needs when presented with a prenatal diagnosis of foetal anomaly. Specifically, we were interested in gaining insights into their experiences of being told about the diagnosis, information offered by health professionals, and formal professional psychosocial support.

## Methods

### Study context

Sweden is a multicultural country, where approximately 17% of the population is foreign born [20]. Swedish maternity care offers pregnant women prenatal tests that screen for foetal anomalies. Prenatal screening in early pregnancy aims to assess the likelihood that the foetus has chromosomal anomalies, i.e. combined biochemical markers and ultrasound examination with nuchal translucency measurement as well as non-invasive prenatal test that analyse cell-free DNA. There are regional differences in the availability of these prenatal tests. As an integral part of Swedish maternity care, all pregnant women are offered a second-trimester obstetric ultrasound examination. This ultrasound examination is intended to assess gestational length, localise the placenta, determine the number of foetuses, and screen for foetal structural anomalies. Most accept the offer to undergo the examination and experience the decision to have the obstetric ultrasound easy to reach [21].

When a foetal anomaly is identified, pregnant women have the option to terminate the pregnancy. Swedish legislation allows women to terminate their pregnancy up until 18 completed weeks of gestation, and in later gestation after approval from the National Board of Health and Welfare [22]. In clinical practice, few induced abortions are performed after 22 gestational weeks. If the woman decides to terminate the pregnancy, a social-worker assists with the application to the National Board of Health and Welfare and offers psychosocial support. Follow-up visits are also offered at the foetal medicine unit following pregnancy termination. If the pregnancy is continued, follow-up visits are offered at the foetal medicine unit, to monitor the progression of the anomaly, optimise the planning of prenatal management, and prepare the woman/couple for the birth. Expectant parents are routinely offered professional psychosocial support at the units.

### Data collection

An Institutional Review Board approved the present study: The Regional Ethical Committee in Uppsala, Sweden (approval number 2014/504). To be eligible for inclusion, participants needed to require interpreter services to understand information from health professionals at the time of diagnosis, and be able to read and write in either Arabic or Sorani.

In August 2015, emails were sent to all Arabic and Kurdish non-profit associations and general women’s associations (*n* = 26) listed on the website of a Swedish research and documentation centre about immigrants, refugees and racism in Sweden. The email contained a request to post a message on the association’s website about the study. One week later, the associations were contacted by telephone. In total, we made contact with ten associations and provided oral information about the study.

Potential participants were invited to participate via a message on the associations’ websites. The message included a link to a web-based tool with information about the study and a question about consent to participate in the study. The information included the aim of the study, that participation was anonymous and completely voluntary, and that they could decide to discontinue their participation without a need for explaining why. Potential participants were informed that the results of the study will be published in scientific journals and that individual authentic quotes may be used in the articles. Information was given that only the researchers involved in the study would have access to the data. Respondents gave their consent to participate by replying “yes” to the following question: “Do you consent to participate in the study?”. Thereafter, the respondent got access to all the questions in the survey. If preferred, respondents could print out hard copy of the questions, which they then mailed to the last author’s workplace. The participants were encouraged to write freely and as much or as little as they desired.

The survey included background questions regarding sex, age, country of birth, educational level, number of years living in Sweden and type of foetal anomaly diagnosed. Two open-ended questions that addressed the aim of the study were included: ‘*Would you please describe what it was like for you to receive the news about the foetal anomaly’* and ‘*Would you please describe how you experienced the information and support from health professionals’* (Additional file 1).

### Participant characteristics

Six women with experience of a prenatal diagnosis sent responses via postal services (*n* = 5) and e-mail (*n* = 1). Three responses were in Arabic and three in Sorani. The ages ranged between 22 and 47 years, and countries of birth were Iraq (*n* = 4), Jordan (*n* = 1) and Morocco (n = 1). The respondents had lived in Sweden between 3 and 22 years. Their educational levels were upper-secondary school (*n* = 3) and university/college (n = 3). The foetal diagnoses were trisomy 21 (*n* = 2), congenital heart defect (*n* = 1), brain defect (n = 1), multiple anomalies (n = 1) and kidney/urinary tract defect (n = 1). The time from diagnosis ranged between 0 and 3 years. Two respondents terminated the pregnancy following the diagnosis.

### Analysis

The responses were translated to Swedish by the second author and subjected to qualitative content analysis, a method to discover patterns in written data, inspired by the outline presented by Graneheim and Lundman [23]. Meaning units were identified, defined as words, sentences, and paragraphs related to the aim of the study. These meaning units were condensed and assigned a descriptive code, defined as a label of its core content. The codes were placed in themes that portrayed the experiences described in the responses. The second and first author performed two separate analyses, and their findings were then compared and scrutinised until consensus was reached among all three authors.

### Findings

The analysis resulted in three themes: (1) a hurricane of emotions, (2) trying to understand the situation though information in an unfamiliar language, and (3) being cared for in a country with accessible obstetric care and legal induced abortion.

### A hurricane of emotions

The respondents described that they had been joyful over their pregnancies, that they had longed for parenthood, and that they expected the ultrasound examination to show normal findings. One wrote that she was prepared for the ultrasound to indicate trisomy 21, and greatly appreciated the routine obstetric ultrasound examination to screen for foetal anomalies. For others, the news of the anomaly came as a completely unexpected emotional shock.

*It came as a shock to us.*

*It became clear that the foetus had Down syndrome. News we were prepared for because I was 44 years old.*

The respondents mentioned that it was difficult for them to express their emotional state in words. Feelings such as sadness, loneliness, and sorrow were described when faced with the prenatal diagnosis. The news of the foetal anomaly was described as the worst possible news that an expectant mother could receive, equated to a great hurricane of emotions. One wrote that she would rather have died than been presented with the news of the anomaly. Another experienced great difficulty sleeping following the diagnosis, while one described worries and fears about her future. The respondents also expressed fatalistic thoughts in the sense that their future was written in destiny and that it was their expected child’s fate to die, as decided by God.

*I could not think of anything worse than to hear about the news that I just received [...] I would rather have died than find out such news about the foetus.*

*It was my first pregnancy and I was so happy. My husband and I had tried to get pregnant. When I received the news, it felt like a hurricane came over me. We expected a joyous result [of the ultrasound examination]. But, what we instead received was heavy news that washed over us in indescribable silence and tears.*

### Trying to understand the situation though information in an unfamiliar language

The respondents called attention to the importance of sufficient and comprehensible information following the diagnosis. They greatly appreciated the opportunities they were given to ask questions and receive detailed information from various health professionals. However, language barriers hindered information comprehension and informed decision-making regarding the alternatives of continuing or terminating the pregnancy. Health professionals had used complex medical terminology, which made it difficult to understand the severity of the anomalies. Moreover, the respondents mentioned that it had been difficult for the interpreters to translate medical terminology to their mother tongue, which made them question the accuracy of the interpretation services.

*Neither I nor my partner could speak Swedish well enough. But, later a person came who translated for us. However, I’m afraid we didn’t get all the information that was given as I think that the person who was translating didn’t understand medical terms.*

Some expressed that they might have made a different decision concerning whether to continue or terminate the pregnancy, if they had comprehended the information offered by health professionals with more clarity.

*Honestly, I didn’t understand how seriously malformed the child was because I didn’t master the language well enough. The information from the staff was not easy to understand either. If I had understood [correctly], I would have taken a different decision. The staff used many words and phrases that were difficult to understand at a time when I was feeling grief for my child.*

The information about termination of the pregnancy offered by health professionals was described as insufficient. One woman expressed that the induced abortion had been a very exhausting experience, likened to childbirth, with complications involving considerable pain and bleeding. This respondent indicated that she felt insufficiently informed during the abortion process, in particular what happened to the foetus following the expulsion.

*I felt less well informed during the actual termination. They should have provided me with an answer regarding where the foetus went.*

### Being cared for in a country with accessible obstetric care and legal induced abortion

Despite experiencing instances of insufficient information, the respondents highly valued the respectful and empathic support they received following the diagnosis and during the pregnancy termination. The psychological support they received from nurse-midwives and social workers eased their emotionally difficult situation and helped them process their experiences. Moreover, the respondents expressed gratitude for being in a country like Sweden, where they were offered obstetric ultrasound examinations to screen for foetal anomalies and were able to decide for themselves whether to continue or terminate the pregnancy. The respondents compared the care they were given to that provided in their native countries, and valued the care they were provided following the diagnosis.

*I’m really surprised how we were treated by hospital staff and how they behaved. This was the first time I had ever gotten that kind of help and respect. It was very different from in my native country.*

## Discussion

By using an open-ended approach with web-based recruitment and anonymous data collection, we aimed to explore experiences among immigrants presented with a prenatal diagnosis. The findings show that the respondents were faced with an unexpected personal tragedy that involved various emotional difficulties. They appreciated the opportunity to screen for foetal anomalies during pregnancy and the legal option of terminating the pregnancy. Moreover, they valued the respectful and empathic care they were provided following the diagnosis and during pregnancy termination. However, their information uptake was hindered by language barriers and further complicated by insufficient communication via interpreters. The respondents who terminated their pregnancy described emotional strain during the procedure and insufficient information about the induced abortion, calling attention to the specific need for information and support.

We did not ask about the women’s religious beliefs. However, some narratives included religious tones. The literature underscores the role of religion in decision-making and the importance of health professionals having sufficient knowledge about the impact that religious beliefs may have on the decision [9, 24–26]. Thus, relevant information about the foetal anomaly needs to be discussed based on the woman’s personal values and beliefs [25]. Nevertheless, emotional distress following the diagnosis may outweigh social and religious aspects following a foetal diagnosis [6], calling attention to the importance of adequate psychosocial care for expectant parents faced with a diagnosis. In Sweden, professional psychosocial support is integrated in foetal medicine care and is routinely offered to all faced with a prenatal diagnosis. This may explain why women in this study were satisfied with the care provided, even though they did not fully comprehend the medical information.

Communication difficulties were described, which may be related to a failure to recognise low health literacy [27], i.e. the degree to which individuals have the capacity to obtain, process, and understand health information and services needed to make appropriate health decisions [28]. Although low health literacy is prevalent in the general population, at-risk subgroups include immigrants, low-income populations, and ethnic minorities [29, 30]. Health literacy is a crucial factor for empowerment as low literacy is a primary factor behind health disparities [30–32]. Strategies that aim to improve the health literacy of immigrants include the use of health care interpreters, patient navigators, and other means of communication such as pictograms, images, photographs, audio, and videos [30]. Such interventions have the potential to improve prenatal counselling for pregnant immigrants, and future research should investigate this further.

Different strategies for dealing with language barriers in health care are suggested in the literature, including offering supplemental written information in different languages [33], using translating applications [34] and communication with the aid of medical interpreters [35]. Combining face-to-face counselling with supplemental written information has been shown to enhance knowledge about prenatal screening [36].Translating apps are potentially useful services that are available around-the-clock. However, they require sufficient ability to read and do not translate meaning [34]. Medical interpreters, on the other hand, are educated and guided by a set of medical interpreting standards designed to ensure an accurate and clear line of communication. To achieve high quality in medical interpreting, a total of nine different standards need to be sufficiently met, namely accuracy, advocacy, confidentiality, cultural awareness, impartiality, professional development, professionalism, respect, and role boundaries [37]. However, problems with the accuracy of interpretation have been reported in this as well as other studies [38], illustrating the need for interpreters trained in advanced medical terminology. Communication with health professionals is a central issue for immigrant women [39], and both adequate information via an interpreter and improved training of health professionals are important aspects of the care of these women [40]. However, in line with previous studies in other settings [41], the findings of this study indicate that use of professional interpreters is not optimal in the care of women faced with a prenatal diagnosis. It has been put forward that health professional’s use of interpreters is hindered by interpreter accessibility and availability as well as lack of time [42]. Other studies point out the overlapping roles between health professionals and interpreters as barriers to interpreter use [38]. Taken together, we argue that use of interpreters is a complex issue that requires special attention so that sufficient information uptake is promoted following the diagnosis.

The findings indicate that informed decisions to continue or terminate the pregnancy are not always reached among immigrant women in need of interpreter services following a prenatal diagnosis. Among native speakers, the decision is considered difficult [7] and is characterised as a chosen loss with emotional distress and perinatal grief [43]. Informed decisions could potentially be hindered by language barriers, as indicated by the findings of this study. These findings illustrate the importance of adequate communication to meet the needs of these women. Medically induced second-trimester abortions are procedures associated with considerable physical pain and emotional distress as identified in research [12]. Moreover, preparatory information is an acknowledged key aspect for high quality in abortion care [44], calling attention to the importance of adequate information before undergoing the procedure. However, research suggests that women undergoing induced second-trimester abortions describe insufficient information and unpreparedness [12]. Our findings confirm that immigrants with interpreter needs experience similar circumstances. The findings illustrate that health professionals need to acknowledge the need for clear and comprehensible information about pregnancy termination in order to promote preparedness for the abortion process. Steps may be needed to ensure that information about medically induced abortions transcends language barriers so that women are provided equal opportunities to receive preparatory information. The findings of this study highlight the need for more descriptive studies that investigate decision-making and preparedness for induced abortion among immigrants faced with a prenatal diagnosis.

This study contributes to the knowledge regarding immigrants’ experiences of information and support following a prenatal diagnosis of a foetal anomaly. The anonymous web-based data collection method made it possible to explore a phenomenon without the direct influence of an interviewer. Written material may contain more intimate details than interviews [45], and it is possible that the approach encouraged the respondents to write freely about their experiences following the diagnosis. This may be especially valuable in the context of this study since induced abortions carry an identified social stigma [46]. However, it is important to bear in mind that the method used to collect data required sufficient reading and writing skills. The UNESCO Institute for Statistics concludes that there are significant variations in literacy rates between world regions [47] and that the majority (61%) of the world’s illiterate adults are women [48]. We cannot make any claims as to whether the results from this study apply to female immigrants who are unable to read and write. Future studies should strive to recruit these women. In addition, finding feasible ways to improve immigrants’ health literacy skills and reduce the negative effects of low health literacy on health outcomes are warranted [49]. As a mean towards this end, cooperation between policymakers, clinicians and other stakeholders is needed.

There are additional limitations of this study that should be considered when interpreting the findings. First, the design of the data collection made it impossible to ask follow-up questions, which may have limited the possibility of fully exploring the respondents’ experiences. However, we encouraged respondents to write freely and as much as they desired. Secondly, we did not receive any responses from males, and therefore, the transferability may be limited to immigrant women. The respondents were women of different ages, educational levels and with Arabic or Sorani as their native language. If immigrants with other native languages had responded, it is possible that there would have been other findings. More research is needed to explore the experiences of immigrants of various backgrounds and to investigate how health professionals can most appropriately promote information uptake, regardless of whether the pregnancy is continued or terminated. Thirdly, we received answers from six women. We acknowledge that this is a limited number of respondents, recruited through means of convenience sampling. This was an exploratory qualitative study, and the findings should be viewed as hypothesis-generating, and a potential guide towards future directions in research.

## Conclusion

Immigrant women experience an unexpected personal tragedy when faced with a prenatal diagnosis of foetal anomaly, and emphasise the importance of respectful and empathic support. The experiences of insufficient and incomprehensible information call attention to the importance of tailored approaches to suit individual needs. Inaccurate interpretation services may further complicate information uptake, and the findings emphasise the need for adequate interpretation services to ensure informed decisions regarding whether to continue or terminate the pregnancy. There is a need for more descriptive studies that investigate decision-making and preparedness for induced abortion among immigrants faced with a prenatal diagnosis.

## Additional file

Additional file 1: Questions in the web-based tool. The file includes all questions in the web-based tool that the respondents answered. (PDF 42 kb)

## References

[1] Frøslev-Friis C, Hjort-Pedersen K, Henriques CU, Krogh LN, Garne E (2011). Improved prenatal detection of chromosomal anomalies. Dan Med Bull.

[2] Dolk H, Loane M, Garne E (2010). The prevalence of congenital anomalies in Europe. Adv Exp Med Biol.

[3] Garcia J, Bricker L, Henderson J, Martin M-A, Mugford M, Nielson J (2002). Women’s views of pregnancy ultrasound: a systematic review. Birth..

[4] Wool C (2011). Systematic review of the literature: parental outcomes after diagnosis of fetal anomaly. Adv Neonatal Care.

[5] Abi Tayeh G, Jouannic J-M, Mansour F, Kesrouani A, Attieh E (2018). Complexity of consenting for medical termination of pregnancy: prospective and longitudinal study in Paris. BMC Med Ethics.

[6] Gesser-Edelsburg A, Shahbari NAE (2017). Decision-making on terminating pregnancy for Muslim Arab women pregnant with fetuses with congenital anomalies: maternal affect and doctor-patient communication. Reprod Health.

[7] Asplin N, Wessel H, Marions L, Georgsson Öhman S (2013). Pregnant women’s perspectives on decision-making when a fetal malformation is detected by ultrasound examination. Sex Reprod Healthc.

[8] Souka AP, Michalitsi VD, Skentou H, Euripioti H, Papadopoulos GK, Kassanos D (2010). Attitudes of pregnant women regarding termination of pregnancy for fetal abnormality. Prenat Diagn.

[9] Shaw A (2012). “They say Islam has a solution for everything, so why are there no guidelines for this?” ethical dilemmas associated with the births and deaths of infants with fatal abnormalities from a small sample of Pakistani Muslim couples in Britain. Bioethics..

[10] Ruschel P, Zielinsky P, Grings C, Pimentel J, Azevedo L, Paniagua R (2014). Maternal-fetal attachment and prenatal diagnosis of heart disease. Eur J Obstet Gynecol Reprod Biol.

[11] Brosig CL, Whitstone BN, Frommelt MA, Frisbee SJ, Leuthner SR (2007). Psychological distress in parents of children with severe congenital heart disease: the impact of prenatal versus postnatal diagnosis. J Perinatol.

[12] Andersson I-M, Christensson K, Gemzell-Danielsson K (2014). Experiences, feelings and thoughts of women undergoing second trimester medical termination of pregnancy. PLoS One.

[13] Korenromp MJ, Page-Christiaens GCML, van den Bout J, Mulder EJH, Visser GHA (2009). Adjustment to termination of pregnancy for fetal anomaly: a longitudinal study in women at 4, 8, and 16 months. Am J Obstet Gynecol.

[14] Carlsson T, Bergman G, Wadensten B, Mattsson E (2016). Experiences of informational needs and received information following a prenatal diagnosis of congenital heart defect. Prenat Diagn.

[15] Bratt E-L, Järvholm S, Ekman-Joelsson B-M, Mattson L-Å, Mellander M (2015). Parent’s experiences of counselling and their need for support following a prenatal diagnosis of congenital heart disease - a qualitative study in a Swedish context. BMC Pregnancy Childbirth.

[16] Lafarge C, Mitchell K, Fox P (2014). Termination of pregnancy for fetal abnormality: a meta-ethnography of women’s experiences. Reprod Health Matters.

[17] United Nations. International migration report 2015. 2016. http://www.who.int/migrants/publications/MigrationReport2015.pdf?ua=1. Accessed 30 Apr 2019.

[18] Pimentel VM, Eckardt MJ (2014). More than interpreters needed: the specialized care of the immigrant pregnant patient. Obstet Gynecol Surv.

[19] Small R, Roth C, Raval M, Shafiei T, Korfker D, Heaman M (2014). Immigrant and non-immigrant women’s experiences of maternity care: a systematic and comparative review of studies in five countries. BMC Pregnancy Childbirth..

[20] Sweden S (2015). Statistikdatabasen [statistics database].

[21] Crang-Svalenius E, Dykes A-K, Jörgensen C (1998). Factors influencing informed choice of prenatal diagnosis: women’s feelings and attitudes. Fetal Diagn Ther.

[22] Swedish Riksdag. Abortlag (1974:595) [abortion act (1974:595)]. 1974. http://www.riksdagen.se/sv/dokument-lagar/dokument/svensk-forfattningssamling/abortlag-1974595_sfs-1974-595. Accessed 30 Apr 2019.

[23] Graneheim UH, Lundman B (2004). Qualitative content analysis in nursing research: concepts, procedures and measures to achieve trustworthiness. Nurse Educ Today.

[24] Noble A, Engelhardt K, Newsome-Wicks M, Woloski-Wruble AC (2009). Cultural competence and ethnic attitudes of midwives concerning Jewish couples. J Obstet Gynecol Neonatal Nurs JOGNN.

[25] Gitsels-van der Wal JT, Manniën J, Gitsels LA, Reinders HS, Verhoeven PS, Ghaly MM (2014). Prenatal screening for congenital anomalies: exploring midwives’ perceptions of counseling clients with religious backgrounds. BMC Pregnancy Childbirth..

[26] Stephens M, Jordens CFC, Kerridge IH, Ankeny RA (2010). Religious perspectives on abortion and a secular response. J Relig Health.

[27] Groene RO (2014). Is health literacy addressed in the medical education of general practitioners? The 2nd European health literacy conference.

[28] Institute of Medicine (US) Committee on Health Literacy (2004). Health literacy: a prescription to end confusion. Washington (DC): national academies press.

[29] Berkman ND, Sheridan SL, Donahue KE, Halpern DJ, Viera A, Crotty K (2011). Health literacy interventions and outcomes: an updated systematic review. Rockville.

[30] Kickbusch I, Pelikan JM, Apfel F, Tsouros AD. Health literacy. The solid facts: WHO Regional Office for Europe; 2013. http://www.euro.who.int/__data/assets/pdf_file/0008/190655/e96854.pdf

[31] Parker R, Ratzan SC (2010). Health literacy: a second decade of distinction for Americans. J Health Commun.

[32] World Health Organization. Achieving health equity: from root causes for fair outcomes. 2007. http://www.who.int/social_determinants/thecommission/interimstatement/en/. Accessed 30 Apr 2019.

[33] Higginbottom GM, Safipour J, Yohani S, O’Brien B, Mumtaz Z, Paton P (2016). An ethnographic investigation of the maternity healthcare experience of immigrants in rural and urban Alberta, Canada. BMC Pregnancy Childbirth..

[34] Sarfraz S, Wacogne ID. Fifteen-minute consultation: how to use an interpreter in a medical consultation. Arch Dis Child Educ Pract Ed. 2018. 10.1136/archdischild-2018-315989 30442675

[35] Purnell LD (2009). Guide to culturally competent health care.

[36] Yeniceri EN, Kasap B, Akbaba E, Akin MN, Sariyildiz B, Kucuk M (2017). Knowledge and attitude changes of pregnant women regarding prenatal screening and diagnostic tests after counselling. Clin Exp Obstet Gynecol.

[37] National Council of Interpreting in Health Care. The national standards of practice for interpreters in health care. 2005. https://www.ncihc.org/assets/documents/publications/NCIHC%20National%20Standards%20of%20Practice.pdf. Accessed 10 Jan 2019.

[38] Hsieh E (2007). Interpreters as co-diagnosticians: overlapping roles and services between providers and interpreters. Soc Sci Med 1982.

[39] Delvaux T, Buekens P, Godin I, Boutsen M (2001). Barriers to prenatal care in Europe. Am J Prev Med.

[40] Esscher A, Binder-Finnema P, Bødker B, Högberg U, Mulic-Lutvica A, Essén B (2014). Suboptimal care and maternal mortality among foreign-born women in Sweden: maternal death audit with application of the “migration three delays” model. BMC Pregnancy Childbirth..

[41] Gerrish K (2001). The nature and effect of communication difficulties arising from interactions between district nurses and south Asian patients and their carers. J Adv Nurs.

[42] Ramirez D, Engel KG, Tang TS (2008). Language interpreter utilization in the emergency department setting: a clinical review. J Health Care Poor Underserved.

[43] Sandelowski M, Barroso J (2005). The travesty of choosing after positive prenatal diagnosis. J Obstet Gynecol Neonatal Nurs.

[44] Dennis A, Blanchard K, Bessenaar T (2017). Identifying indicators for quality abortion care: a systematic literature review. J Fam Plann Reprod Health Care.

[45] Seale C, Charteris-Black J, MacFarlane A, McPherson A (2010). Interviews and internet forums: a comparison of two sources of qualitative data. Qual Health Res.

[46] Astbury-Ward E, Parry O, Carnwell R (2012). Stigma, abortion, and disclosure--findings from a qualitative study. J Sex Med.

[47] UIS. Education: literacy rate. data.uis.unesco.org. Accessed 27 Dec 2018.

[48] UNESCO (2006). Education for all global monitoring report.

[49] Fernández-Gutiérrez M, Bas-Sarmiento P, Albar-Marín MJ, Paloma-Castro O, Romero-Sánchez JM (2018). Health literacy interventions for immigrant populations: a systematic review. Int Nurs Rev.

