# Supplementary material for: Written narratives from immigrants following a prenatal diagnosis: qualitative exploratory study
Source: BMC Pregnancy Childbirth. 2019 May 6;19:154. doi: 10.1186/s12884-019-2292-9 (PMC6501294; doi:10.1186/s12884-019-2292-9)
Supplement: Supplementary file 1 — Questions in the web-based tool. The file includes all questions in the web-based tool that the respondents answered. (PDF 42 kb) [file 12884_2019_2292_MOESM1_ESM.pdf]

Additional File 1. Questions in the web-based tool.

1. What is your gender?

2. What is your age?

3. What country are you from?

4. How many years have you lived in Sweden?

5. What is your highest education?

6. What experience do you have of a detected foetal anomaly during pregnancy?
(Pregnant or partner to pregnant)

7. What type of foetal anomaly was discovered during your pregnancy?

8. Would you please describe what it was like for you to receive the news about the foetal anomaly.

9. Would you please describe how you experienced the information and support from health professionals
